# Magnesium sulphate at 30 to 34 weeks’ gestational age: neuroprotection trial (MAGENTA) - study protocol

**DOI:** 10.1186/1471-2393-13-91

**Published:** 2013-04-09

**Authors:** Caroline A Crowther, Philippa F Middleton, Dominic Wilkinson, Pat Ashwood, Ross Haslam

**Affiliations:** 1 Australian Research Centre for Health of Women and Babies (ARCH), The Robinson Institute, The University of Adelaide, Adelaide, Australia; 2 Liggins Institute, The University of Auckland, Auckland, New Zealand; 3 Department of Neonatal Medicine, The Women’s and Children’s Hospital, Adelaide, South Australia, Australia

**Keywords:** Magnesium sulphate, Neuroprotection, Preterm birth, Randomised controlled trial, Cerebral palsy

## Abstract

**Background:**

Magnesium sulphate is currently recommended for neuroprotection of preterm infants for women at risk of preterm birth at less than 30 weeks’ gestation, based on high quality evidence of benefit. However there remains uncertainty as to whether these benefits apply at higher gestational ages.

The aim of this randomised controlled trial is to assess whether giving magnesium sulphate compared with placebo to women immediately prior to preterm birth between 30 and 34 weeks’ gestation reduces the risk of death or cerebral palsy in their children at two years’ corrected age.

**Methods/design:**

***Design:*** Randomised, multicentre, placebo controlled trial.

***Inclusion criteria:*** Women, giving informed consent, at risk of preterm birth between 30 to 34 weeks’ gestation, where birth is planned or definitely expected within 24 hours, with a singleton or twin pregnancy and no contraindications to the use of magnesium sulphate.

***Trial entry & randomisation:*** Eligible women will be randomly allocated to receive either magnesium sulphate or placebo.

***Treatment groups:*** Women in the magnesium sulphate group will be administered 50 ml of a 100 ml infusion bag containing 8 g magnesium sulphate heptahydrate [16 mmol magnesium ions]. Women in the placebo group will be administered 50 ml of a 100 ml infusion bag containing isotonic sodium chloride solution (0.9%). Both treatments will be administered through a dedicated IV infusion line over 30 minutes.

***Primary study outcome:*** Death or cerebral palsy measured in children at two years’ corrected age.

***Sample size:*** 1676 children are required to detect a decrease in the combined outcome of death or cerebral palsy, from 9.6% with placebo to 5.4% with magnesium sulphate (two-sided alpha 0.05, 80% power, 5% loss to follow up, design effect 1.2).

**Discussion:**

Given the magnitude of the protective effect in the systematic review, the ongoing uncertainty about benefits at later gestational ages, the serious health and cost consequences of cerebral palsy for the child, family and society, a trial of magnesium sulphate for women at risk of preterm birth between 30 to 34 weeks’ gestation is both important and relevant for clinical practice globally.

**Trial registration:**

Australian New Zealand Clinical Trials Registry - ACTRN12611000491965

## Background

Babies born preterm have a higher chance of dying in the first few weeks of life than those born at term [1]. Babies who survive have a greater risk of neurologic impairments, such as cerebral palsy, blindness, deafness, or cognitive dysfunction, and a greater risk of substantial disability as a result of these neurologic impairments [2-4]. The social and economic long-term costs are considerable [5].

Cerebral palsy is a term which includes a number of different diseases or conditions that can arise at any time during brain development. It involves a disorder of movement or posture, or both, and a disorder of motor function that is permanent but may change over time [6]. The cerebral palsies remain the most frequent cause of severe motor disability in childhood with a background prevalence of two per thousand live births [6]. Most affected children (92%) survive to 20 years or later, yielding a substantial burden of illness into adulthood [7].

Very preterm birth (less than 34 weeks) is the principal risk factor for cerebral palsy [8,9], responsible for 17% to 32% of all cases of cerebral palsy. The latest Australian Cerebral Palsy Register Report (2009) shows that approximately 45% of all cases of cerebral palsy are associated with preterm birth [10]. Whilst the highest risks are for extremely preterm infants [3], babies born between 30 and 33 completed weeks’ gestation still have significant risks [11] with the risk of cerebral palsy being up to eight times more likely than babies born at term [4]. Moderate prematurity is responsible for as many cases of cerebral palsy as extreme prematurity [10].

At present there is no cure for cerebral palsy, which makes effective preventative interventions of paramount importance. Prevention of cerebral palsy has been identified by consumers, clinicians and researchers as a top priority for research by the Australian Cerebral Palsy Institute [12]. To reduce the impact of cerebral palsy from preterm birth, efforts must be focused on primary prevention.

### Observational studies on the effect of antenatal magnesium sulphate on neurodevelopment

A landmark case–control study 15 years ago described the association of exposure to antenatal magnesium sulphate with a dramatic reduction in the risk of cerebral palsy (odds ratio (OR) 0.14; 95% confidence interval (CI) 0.05 to 0.51) [13]. Other observational studies support a reduction in cerebral palsy in preterm babies after antenatal magnesium sulphate [14-16] and some a reduction in the risk of intraventricular hemorrhage (IVH) [16,17] and perinatal mortality [18]. However, not all studies report benefit for antenatal magnesium sulphate on the risk of IVH [19-21], cerebral palsy [20,22,23] or perinatal mortality [19].

### Biological plausibility for use of magnesium sulphate for fetal and infant neuroprotection

In humans, magnesium is essential for key cellular processes, including glycolysis, oxidative phosphorylation, protein synthesis, DNA and RNA aggregation and maintenance of plasma membrane integrity [24,25]. Magnesium favourably affects mechanisms implicated in cell death by decreasing proinflammatory cytokines or free radicals produced during hypoxic-ischaemic reperfusion and inflammatory diseases of pregnancy [26,27]. Magnesium prevents excitotoxic calcium-induced injury [28], by a non-competitive voltage-dependent inhibition of the N-methyl-D-aspartate receptor to glutamate reducing calcium entry into the cell [29]. The fetal and neonatal brain seems more susceptible to glutamate damage. Consequently, blocking glutamate receptors through agents such as magnesium sulphate may reduce the risk of injury in the perinatal period. Magnesium has some beneficial haemodynamic effects including stabilising blood pressure during the first two days of life in preterm neonates [30], and may increase cerebral blood flow by reducing constriction of the cerebral arteries [31]. Transplacental transfer of magnesium is rapid with magnesium concentrations increased in fetal serum within one hour of maternal intravenous administration [32].

### Maternal and neonatal adverse effects and side effects of magnesium sulphate

The best available evidence about potential maternal harms from antenatal magnesium sulphate administration comes from the four Cochrane reviews that compare magnesium sulphate with placebo or no treatment [33-36]. Magnesium sulphate, by its peripheral vasodilator effects when infused intravenously, produces a sensation of warmth and flushing. Reported maternal side-effects, related to dosage and speed of infusion, include nausea, vomiting, headache and palpitations. Hypotension and respiratory depression are more severe recognised risks. Magnesium sulphate acts as a neuromuscular blocking agent that causes abolition of tendon reflexes [37]. Magnesium could aggravate the cardiovascular or neuromuscular side-effects of other drugs such as betamimetics, calcium-channel blockers and gentamicin [38,39]. Infusion to concentrations above the recommended therapeutic range can have life threatening consequences for the women that include respiratory arrest and cardiac arrest leading to death [40]. For the neonate, hypermagnesaemia can lead to hyporeflexia, poor sucking, and, rarely, respiratory depression needing assisted ventilation [41,42].

### How antenatal magnesium sulphate can reduce the burden of being born preterm

#### Systematic review of randomized trials of magnesium sulphate for neonatal neuroprotection

The updated Cochrane systematic review to assess the use of antenatal magnesium sulphate for women at risk of preterm birth [33] included four trials (4446 babies) where magnesium sulphate had been given specifically for neuroprotection of the fetus; two from the US; the MagNet Trial [43] and the BEAM Trial [44], one from Australia and New Zealand; the ACTOMgSO_4_ Trial [45], one from France; the PreMag Trial [46]. There was diversity in the inclusion and exclusion criteria for the four included trials with wide variation in gestational age, reasons women were at risk of preterm birth and time of treatment prior to expected preterm birth [33]. All trials used intravenous magnesium sulphate although the dose used, whether a maintenance infusion was given and whether treatment could be repeated varied between trials.

### Results of the meta-analysis of the Cochrane systematic review

The combined outcome of death or cerebral palsy or cerebral palsy alone showed significant reductions where women who were at risk of preterm birth were given magnesium sulphate antenatally with the intent of providing neuroprotection (Table 1). The review showed that 63 babies (95% CI 44 to 155) need to be treated with magnesium sulphate for one baby to avoid cerebral palsy. The corresponding number needed to treat to benefit (NNTB) for combined death or cerebral palsy was 42 babies, 95% CI 24 to 346.

**Table 1:** **Magnesium sulphate vs placebo/no treatment: primary outcomes**[33]

**Primary outcomes**	**RR, 95% CI**	**Number of trials; participants**
**Death or cerebral palsy**	0.85, 0.74 to 0.98*	four trials; 4446 infants
**Death (fetal and later)**	0.95, 0.80 to 1.12	four trials; 4446 infants
**Cerebral palsy**	0.71, 0.55 to 0.91*	four trials; 4446 infants
**Any neurological impairment**	1.03, 0.87 to 1.21	one trial; 1255 infants
**Death or substantial gross motor dysfunction**	0.84, 0.71 to 1.00	three trials; 4387 infants

*Significantly in favour of magnesium sulphate.

### Is there clinical evidence for the role of antenatal magnesium sulphate for neuroprotection of the fetus, infant and child prior to preterm birth at 30 to 34 weeks gestation?

The Australian and New Zealand Bi-national Clinical Practice Guidelines on the use of antenatal magnesium sulphate prior to preterm birth for neuroprotection of the fetus, infant and child summarise the evidence available from the four individual neuroprotective intent trials within the Doyle 2009 Cochrane Review [33] that consider gestational age at trial entry and effect of antenatal magnesium sulphate [47]. All women in the four included trials were given magnesium sulphate before 34 weeks’ gestation. In Rouse 2008, all women were less than 32 weeks at trial entry with the majority (68% of trial participants) less than 30 weeks gestation [44]. Subgroup analyses for women at different gestational ages were possible for women with a gestational age of less than 34 weeks, less than 33 weeks, less than 32 weeks and less than 30 weeks. However there was only one trial within each subgroup available for analysis and the results are inconclusive due to small sample sizes (Table 2).

**Table 2:** **Results of primary outcomes by gestational age subgroup**[33]

**Trial**	**N**	**Eligible (GA wks)**	**DEATH or CP**	**CP**	**DEATH**
			**RR, 95% CI**	**RR, 95% CI**	**RR, 95% CI**
MagNet^ǂ ^[43]	59	>24 to <34	4.83, 0.60 to 38.90	6.77, 0.37 to 125.65	1.93, 0.19 to 20.18
Marret[46]	688	viable to <33	0.80, 0.58 to 1.10	0.70, 0.41 to 1.19	0.85, 0.55 to 1.32
Rouse[44]	2444	24 to <32	0.90, 0.73 to 1.10	0.59, 0.40 to 0.85*	1.13, 0.87 to 1.48
Crowther[45]	1255	<30	0.82, 0.66 to 1.02	0.85, 0.55 to 1.31	0.81, 0.62 to 1.05
Overall	4446	viable to <34	0.85, 0.75 to 0.98*	0.71, 0.55 to 0.91*	0.95, 0.80 to 1.12

^ǂ^Neuroprotective arm; CP cerebral palsy; GA gestational age; * statistically significant.

### Summary of CPG evidence statement judgements for gestational age subgroup

The subgroup analyses are from trials with low risk of bias, with results between trials fairly consistent. While the evidence is applicable to the Australian and New Zealand context, generalisability was reduced as the majority of the women (87%) in the largest trial [44] had PPROM and so represent a limited subset of women at risk of preterm birth. Overall clinical impact was judged to be very large (Table 2) but any differences in death and cerebral palsy by gestational age are unclear at present. To minimise the number of women exposed, the Australian and New Zealand clinic practice guideline panel felt it would be prudent to restrict magnesium sulphate administration to the subgroup containing the lowest gestational age (less than 30 weeks).

### Recommendations made by the Australian and New Zealand Bi-National CPG panel for use of antenatal magnesium sulphate

The main clinical recommendation is to use magnesium sulphate for neuroprotection of the fetus, infant and child “in women at risk of early preterm (gestational age is less than 30 weeks), imminent birth (when early preterm birth is planned or definitely expected within 24 hours)” [47].

In recognition of the need for further research, the guideline panel specifically recommended that further randomised trials were needed, comparing antenatal magnesium sulphate with placebo when given to women at risk of preterm birth at 30 weeks’ gestation or more, that assess mortality, cerebral palsy and combined death and cerebral palsy [47].

Preventative strategies that may reduce the risk of cerebral palsy need appropriate evaluation prior to introduction into clinical practice. Given the magnitude of the protective effect in the systematic review, the ongoing uncertainty about benefits at later gestational ages, the serious health and cost consequences of this condition for the child, family and society a trial of magnesium sulphate for women at risk of preterm birth between 30 to 34 weeks’ gestation is both justifiable and needed.

### Aims and objectives of this trial

The aim of this randomised controlled trial is to assess whether giving magnesium sulphate compared with placebo to women immediately prior to preterm birth between 30 and 34 weeks’ gestation reduces the risk of death or cerebral palsy in their children at two years’ corrected age.

### Hypotheses

The primary hypothesis is that antenatal magnesium sulphate compared with placebo given to women at risk of preterm birth between 30 and 34 weeks’ gestation when birth is imminent (defined as planned or definitely expected in the next 24 hours) reduces the risk of death or cerebral palsy in their children at two years’ corrected age.

The secondary hypotheses are that antenatal magnesium sulphate compared with placebo given to women at risk of preterm birth between 30 and 34 weeks’ gestation when birth is imminent (defined as planned or definitely expected in the next 24 hours) above has benefits relating to *the infant/child:* including individual components of the primary outcome (mortality and cerebral palsy), and neonatal and childhood morbidity (including IVH and the other neurosensory disabilities at two years’ corrected age of blindness, deafness or developmental delay). At the same time, it is hypothesized that the intervention does not harm *the mother:* no clinically important effect on mode of birth or maternal morbidity as measured by risk of serious adverse cardiovascular effects of the infusion, side effects of the infusion and postpartum haemorrhage.

## Methods/design

### Ethics statement

Ethics approval was granted by the Children’s Youth and Women’s Health Services Human Research Ethics Committee at the Women’s and Children’s Hospital (REC2303/8/13) and by the local institutional review boards for each centre.

### Study design

Randomised, multicentre, placebo controlled trial.

### Inclusion criteria

Women are eligible for the trial if they are at risk of preterm birth between 30 to 34 weeks’ gestation where birth is planned or definitely expected within 24 hours, have a singleton or twin pregnancy, no contraindications to the use of antenatal magnesium sulphate (respiratory depression, hypotension, renal failure, myasthenia gravis) and give informed, written consent.

### Exclusion criteria

Women are not eligible if they have a higher-order multiple pregnancy, have received antenatal magnesium sulphate in the current pregnancy or if magnesium sulphate therapy is considered essential for the treatment of pre-eclampsia [36].

### Trial entry

Eligible women are given the trial information sheet, counselled by a member of the research team and encouraged to discuss the study with family before consent is sought.

### Study groups

Once all entry details are given and eligibility is confirmed, the woman is randomised by contacting the central telephone randomisation service at the University of Adelaide. Assignment to one of two study groups: either the magnesium sulphate group or the placebo group will be stratified for collaborating centre, gestational age (30 to 31 completed weeks; 32–33 completed weeks’ gestation), and number of fetuses (1 or 2). A Study Number is allocated to the woman corresponding to a treatment pack, each of which looks identical and contains a 100 ml infusion bag.

### Magnesium sulphate study group

Women randomised to the magnesium sulphate study group are administered 50 ml of a 100 ml infusion bag containing 8 g magnesium sulphate heptahydrate [16 mmol magnesium ions] through a dedicated intravenous infusion line over 30 minutes.

### Placebo study group

Women randomised to the placebo study group are administered 50 ml of a 100 ml infusion bag containing isotonic sodium chloride solution (0.9%) through a dedicated intravenous infusion line over 30 minutes.

### Both study groups

The woman’s pulse, blood pressure and respiratory rate are assessed and recorded before the infusion is commenced, fifteen minutes after the infusion has started and at the end of the infusion. Any other monitoring as per the individual obstetric unit protocols for intravenous administration of magnesium sulphate is followed. Magnesium toxicity is unlikely with the regimen recommended in this protocol and serum magnesium concentrations do not need to be routinely measured. There is a potential theoretical interaction between magnesium sulphate and nifedipine of hypotension and neuromuscular blockade effects, although this is seldom reported in clinical practice [38,39].

The trial treatment infusion should be stopped if the respiratory rate decreases more than four breaths per minute below baseline, or is less than 12 breaths per minute; or diastolic blood pressure decreases more than 15 mmHg below baseline level [47]. The infusion may be continued when the respiratory rate or the blood pressure return to the baseline levels. Should hypotension or respiratory depression occur, prompt medical review is recommended. If there is clinical concern over respiratory depression calcium gluconate (1 g [10 ml of 10% solution] can be given slowly via the intravenous route over 10 minutes). Resuscitation and ventilatory support should be immediately available, if needed, during administration of the study medication. At the end of the trial treatment infusion, the section on maternal side effects should be completed on the *Treatment form.*

Care during labour and the postnatal stay will be managed by the obstetric team caring for the woman. Care of the neonate is the responsibility of the attending neonatologist.

### Follow up after birth until the time of primary discharge for both groups

The pregnancy and labour data will be extracted from case notes by the masked research assistant at the collaborating hospitals. The postnatal and neonatal data will be collected similarly after discharge of the mother and baby from hospital.

### Longer term follow up

Women enrolled in the trial will give consent for follow-up of their children from birth until two years’ corrected age at the time of the initial, prenatal recruitment. Mothers of all babies discharged home alive will be contacted by a member of the study team from the coordinating centre at The University of Adelaide by mail soon after hospital discharge and then again when their baby is six, 12 and 18 months corrected age. Mothers will be provided with a reply paid envelope and asked to confirm their current contact details by mail. At each of these times, a member of the study team at the coordinating centre will contact the parents by telephone if a response has not been received in the mail.

### Two years assessments

All surviving children will be formally assessed at two years of age, corrected for prematurity, by a developmental paediatrician and psychologist or other assessor trained to administer the Bayley Scales. All assessors will remain blinded to treatment group assignment. Assessments will be made of health, neurodevelopment, behaviour, growth and blood pressure. This format of assessment has been used in our previous trials [45,48]. Although cerebral palsy may not remain a stable diagnosis before five years of age [49], a diagnosis of severe cerebral palsy (defined below) at two years of age is unlikely to change subsequently [50].

*Gross Motor Function:* The paediatric assessment will include a neurological examination to diagnose cerebral palsy (abnormality of tone with motor dysfunction) and other disability outcomes according to previously reported criteria [51]. Gross Motor Function will be assessed using the Gross Motor Function Classification System (GMFCS) level 0 to 5 [52].

*Psychological Assessments:* The psychological assessment will include the cognitive, motor and language scales of the Third Edition of the Bayley Scales of Infant Development (BSID-III) [53]. This is well-standardised with demonstrated validity and reliability. Psychological test scores will be recorded as standardised scores [derived from raw test scores - mean/standard deviation (SD)]. Children with severe developmental delay who are unable to complete the psychological assessment will be given a standardised score of 4 SD below the mean.

*Behaviour:* The child's caregiver will be asked to complete the child behaviour checklist [54].

*General Health, Health Resource Utilisation, Blood Pressure and Body Size:* A general history and physical examination will determine the presence of any significant chronic illness, and data regarding hospital readmissions will be confirmed, where necessary, from the admitting hospital or doctor. Children will be considered blind if visual acuity in both eyes is worse than 6/60. Children will be considered deaf if their hearing loss is sufficient to require hearing aid(s), or worse.

Blood pressure will be measured and converted to Z-scores relative to American data for blood pressure for age, height and gender in childhood [55].

Questionnaires will be completed by the child's caregiver about any respiratory morbidity, history of illness or injury and use of health services since primary hospitalisation (Parent/Caregiver Questionnaire).

The child's height, weight, and head circumference will be measured in the standard way, and values for the relevant centile, percent of median, and standard deviation scores (Z scores) specific for age and gender will be computed from the British Growth Reference [56].

### Categorisation of neurosensory disability

Children will be considered to have a neurosensory impairment if they have cerebral palsy, GMFCS level 1 to 5, blindness, deafness any of the Bayley Scale scores more than 1 SD below the mean (<−1SD). The neurosensory disabilities imposed by the various neurosensory impairments will be classified as severe, moderate or mild [51] (Table 3).

**Table 3:** **Neurosensory Disability Classifications**[51]

**Severe Disability**	Any severe cerebral palsy (child non-ambulant and likely to remain so; GMFCS level 4 or 5), severe developmental delay (standardised score <−3 SD) or blindness.
**Moderate Disability**	Moderate cerebral palsy (child non-ambulant at 2 years of age but who is likely to ambulate subsequently; GMFCS level 2 or 3), or deafness, or moderate developmental delay (standardised score from −3 SD to <−2 SD).
**Mild Disability**	Mild cerebral palsy (child walking at 2 years of age with only minimal limitation of movement (GMFCS level 1), or suspect developmental delay (standardised score from −2 SD to <−1 SD).
**No Neurosensory Disability**	Children without any neurosensory impairment.

### Primary study endpoints

The primary study endpoint measured in the children at two years’ corrected age is the combined incidence of death or cerebral palsy defined as stillbirths, death of live born infant before hospital discharge or death after hospital discharge before two years’ corrected age; or any cerebral palsy [abnormality of tone with motor dysfunction].

### Secondary study endpoints

#### For the infant

•Health outcomes considered to be important measures of mortality and morbidity prior to primary hospital discharge; (defined as stillbirth and death of liveborn infant before hospital discharge; IVH, severe IVH, cystic PVL, neonatal encephalopathy, neonatal convulsions, proven necrotising enterocolitis, retinopathy of prematurity needing treatment, patent ductus arteriosus needing treatment, respiratory distress syndrome, severity of any neonatal lung disease, chronic lung disease (oxygen dependent at 36 weeks post-menstrual age or 28 days of life if born after 32 weeks gestation), use of respiratory support, airleak requiring drainage, confirmed infection within the first 48 hours, infection after the first 48 hours, body size at birth (weight, length, head circumference) and at discharge home).

•Composite serious health outcome (defined as stillbirth and death of liveborn infant before hospital discharge severe respiratory disease, severe intraventricular haemorrhage (grade 3 & 4); chronic lung disease (oxygen dependent at 36 weeks post-menstrual age or 28 days of life if born after 32 weeks gestation); proven necrotising enterocolitis; severe retinopathy of prematurity (Stage 3 or worse in the better eye); cystic periventricular leukomalacia).

#### For the child

Key health outcomes assessed at two years’ corrected age:

•Individual components of the primary outcome including severity of cerebral palsy (defined as death; cerebral palsy).

•Death or any neurosensory disability (*death* defined as stillbirth, death of live born infant before hospital discharge or death after hospital discharge; and *any neurosensory disabilities* that includes the neurosensory impairments of *cerebral palsy,* [GMFCS level 1 to 5], *blindness* [corrected visual acuity worse than 6/60 in the better eye], *deafness* [hearing loss requiring amplification or worse], and any *developmental delay* defined as standardised score more than 1 SD below the mean (< −1SD).

•Death or major neurosensory disability. Major neurosensory disability includes *severe and moderate disability* (defined as any of: legal blindness, sensorineural deafness requiring hearing aids; moderate or severe cerebral palsy [GMFCS level 2 to 5] or developmental delay/intellectual impairment [standardised score more than two SD below the mean].

•General health of the child (including use of health services since primary hospitalisation), *childhood respiratory morbidity, blood pressure* (Z scores and proportions in hypertensive ranges), *behaviour* as assessed by the Child Behaviour Checklist [54] and body size.

#### For the mother

•Maternal serious adverse cardiovascular and/or respiratory outcome of the infusion (defined as maternal death, cardiac arrest, respiratory arrest).

•Maternal side effects of the infusion (including nausea; vomiting; flushing, infusion arm discomfort; mouth dryness; sweating; dizziness; blurred vision; respiratory rate decreased > four breaths per minute below baseline or <12 breaths per minute; blood pressure decreased > 15 mmHg below baseline level; whether the infusion is discontinued because of side effects).

•Incidence of postpartum haemorrhage, estimated blood loss at birth 500 ml or more; and major postpartum haemorrhage, estimated blood loss 1500 ml or more; mode of birth.

### Sample size

The rate of our primary outcome of death or cerebral palsy at two years’ corrected age in an Australian/New Zealand population between 30 to 34 weeks’ gestation is estimated to be 9.6%, using a predicted mortality rate up to 2 years of age of 4.4% (excluding lethal anomalies) [57] and a predicted rate of cerebral palsy in survivors of 5.2% [46,48].

A trial of 1676 children (838 per group), allowing for a design effect of 1.2 for clustering of babies within mothers and a 5% loss to follow up, with an absolute risk difference of 4.2% [45,46,48] will have 80% power to detect a statistically significant difference at an alpha level of 0.05 (two-tailed) of a decrease in the combined outcome of death or cerebral palsy from 9.6% to 5.4% with magnesium sulphate compared with placebo.

### Analysis and reporting of results

Data will be analysed by a statistician independent of the clinical investigators. Potential confounding variables will comprise socio-demographic variables, such as ethnicity, language spoken at home, family structure, mother’s marital status, social class, and mother’s and father’s education, as well as gender of the baby. Comparisons will be made between treatment groups for the primary and secondary endpoints using an intention to treat approach. Analyses will make adjustments for the stratification variables and for important baseline predictors including gestational age at birth, and reasons for risk of preterm birth. Log binomial regression and linear regression will be used to examine dichotomous and continuous outcomes respectively with results presented as relative risks or differences in means along with 95% confidence intervals. Adjustment till be made for clustering due to multiple births for infant outcomes using generalized estimating equations. *P*-values <0.05 will be considered statistically significant.

## Discussion

The MAGENTA randomised trial assessing the use of antenatal magnesium sulphate in women at risk of preterm birth between 30 to 34 weeks’ gestation for neuroprotection of their infants is important and relevant for clinical practice globally.

## Abbreviations

CI: Confidence interval; GMFCS: Gross motor function classification system; IVH: Intraventricular haemorrhage; NNTB: Number needed to treat to benefit; OR: Odds ratio, PVL, periventricular leukomalacia; SD: Standard deviation.

## Competing interests

The authors declare they have no competing interests. This study is funded through the National Health and Medical Research Council of Australia (NHMRC) in a 5 year project grant (No. 1022968).

## Authors’ contributions

CAC, PFM, DW, PA and RH are all members of the MAGENTA Study Group. The primary investigator of the MAGENTA Study (CAC) wrote the first draft of the MAGENTA protocol and prepared the initial draft. All authors were involved in the development of the design of the study, the protocol development, have commented on all drafts of the protocol, and have read and approved the final draft of the protocol.

## Pre-publication history

The pre-publication history for this paper can be accessed here:

http://www.biomedcentral.com/1471-2393/13/91/prepub
